# Viruses in Laboratory *Drosophila* and Their Impact on Host Gene Expression

**DOI:** 10.3390/v15091849

**Published:** 2023-08-31

**Authors:** Oumie Kuyateh, Darren J. Obbard

**Affiliations:** 1 Institute of Ecology and Evolution, University of Edinburgh, Ashworth Laboratories, Charlotte Auerbach Road, Edinburgh EH9 3FL, UK; darren.obbard@ed.ac.uk; 2 Parasites and Microbes, Wellcome Sanger Institute, Wellcome Genome Campus, Hinxton, Cambridge CB10 1SA, UK

**Keywords:** *Drosophila melanogaster*, virus, immunity, transcriptome, Drosophila C virus, Drosophila A virus, nora virus, galbut virus

## Abstract

*Drosophila melanogaster* has one of the best characterized antiviral immune responses among invertebrates. However, relatively few easily transmitted natural virus isolates are available, and so many *Drosophila* experiments have been performed using artificial infection routes and artificial host–virus combinations. These may not reflect natural infections, especially for subtle phenotypes such as gene expression. Here, to explore the laboratory virus community and to better understand how natural virus infections induce changes in gene expression, we have analysed seven publicly available *D. melanogaster* transcriptomic sequencing datasets that were originally sequenced for projects unrelated to virus infection. We have found ten known viruses—including five that have not been experimentally isolated—but no previously unknown viruses. Our analysis of host gene expression revealed that numerous genes were differentially expressed in flies that were naturally infected with a virus. For example, flies infected with nora virus showed patterns of gene expression consistent with intestinal vacuolization and possible host repair via the upd3 JAK/STAT pathway. We also found marked sex differences in virus-induced differential gene expression. Our results show that natural virus infection in laboratory *Drosophila* does indeed induce detectable changes in gene expression, suggesting that this may form an important background condition for experimental studies in the laboratory.

## 1. Introduction

The antiviral immune response of *Drosophila melanogaster* is among the best characterized of any invertebrate, and antiviral responses in *Drosophila* are mediated by pathways that are conserved across many taxa [1]. These include RNA interference (RNAi), the immune deficiency pathway (Imd), the toll–dorsal pathway (Toll), Janus kinase/signal transducer, activator of transcription pathway (JAK/STAT), autophagy, and the stimulator of interferon genes (STING) signalling cascade [2,3,4,5,6,7,8].

Virus infection may be associated with dramatic changes in gene expression. First, infections trigger host signalling cascades that eventually alter the expression of host immune effector molecules. For example, wild-type *D. melanogaster* infected with Drosophila C Virus (DCV) displays increased expression of genes such *Spaetzle* (*Spz*) and *Drosomycin* (*Drs*) that encode a cytokine and an antimicrobial peptide, respectively, and which are important in the toll pathway [9]. There is also increased expression of the immune-associated genes, CG12780, vir-1, and *listericin* (CG9080) [10]. Second, the expression of non-immune genes changes as a consequence of infection, either through viral manipulation of the host or through the consequences of disease. For example, female flies infected with Drosophila Kallithea Nudivirus display decreased expression of chorion proteins as they cease laying eggs [11].

Much of what is known about the *Drosophila* expression response to viral infection has come from transcriptome profiling studies e.g., [11,12,13,14]. While a minority of studies use natural infection routes, most rely on systemic viral infections established through microinjection (or septic puncture) into the body cavity. However, the infection route of pathogens can substantially affect the outcome of infection, and can trigger different antiviral responses [15,16,17]. This may in part be because injection bypasses immune responses at the natural site of pathogen entry, such as the cuticle, trachea, gut, and reproductive organs. For example, the Toll pathway appears necessary for resistance to oral DCV infection via activation of the transcription factor Dorsal, whereas it is not apparently required in resistance to systemic DCV infection [18]. The route of infection is also important in shaping viral tropism, host response, and pathology. For example, DCV infection is almost 100% lethal when injected but causes only 10–25% mortality when flies are infected orally—even with very high viral titres [18,19]. Remarkably, oral DCV infections at the larval stage are reported to protect flies during reinfection by injection as adults [16]. In contrast, it has been reported that flies injected with a sublethal dose of DCV are not protected against subsequent DCV injection, indicating the importance of oral infection in priming the immune response [20].

A relative lack of natural virus isolates may also limit studies of host–virus interaction using the *Drosophila* model. Such interactions can be highly host-specific [21]; for example, it has been suggested that the Virus protein 1 (VP1) of Drosophila immigrans nora virus is unable to suppress the antiviral RNAi pathway in *D. melanogaster*, whereas it can suppress it in *D. immigrans* [22]. However, many studies in *Drosophila* have used non-native viruses such as Flock House Virus (a beetle virus, e.g., [2]), Sindbis virus (a mosquito alphavirus, e.g., [23]), Invertebrate Iridescent Virus 6 (a moth iridovirus, e.g., [24]), and Cricket Paralysis Virus (a moth dicistrovirus, e.g., [25]).

Despite the fact that metagenomic sequencing of wild *Drosophila* has revealed over 120 naturally occurring fruit fly viruses spanning more than 25 families [26,27,28,29,30], until recently, relatively few natural *Drosophila melanogaster* viruses had available isolates; only the RNA viruses DCV, D. melanogaster Sigmavirus (DmelSV), Drosophila A virus (DAV), Drosophila X virus (DXV), nora virus, and the DNA virus, Drosophila Kallithea nudivirus virus [11,31,32,33]. More recently, galbut virus, La Jolla virus, and Newfield virus have also been isolated [34,35], but these have not yet been widely used in experimental studies.

Some of these viruses are known to occur in laboratory flies and cell culture. The most common viruses reported from laboratory fly stocks include DCV, nora virus, DAV, Newfield virus, DMelSV, and Thika virus [29]. In addition, Drosophila X virus, Drosophila Totivirus, Newfield virus, American Nodavirus, Bloomfield virus, nora virus, DCV, and DAV have all been found laboratory cell cultures [29,36]. The widespread occurrence of such viruses in experimental stocks raises the question of whether changes in gene expression induced by these viruses can impact laboratory experiments. For example, viruses can affect fecundity and shorten development time and lifespan of *Drosophila* [11,37,38,39] and can change fruit fly behaviour and mobility [11,39,40,41], which may negatively impact the interpretability of life history and developmental studies.

Here, we survey seven publicly available adult transcriptome project datasets from laboratory *D. melanogaster* to quantify the prevalence of viruses in experimental studies, and to assess the impact of viruses on patterns of host gene expression. As these infections were incidental and unintended by the original authors, they reflect natural laboratory infection routes and host–virus combinations. The resulting changes in gene expression are suggestive of a reduction in host reproductive investment, and nora-virus-induced gut pathology and host repair. These results imply that background changes in gene expression due to viral infection may be relevant to laboratory experiments.

## 2. Materials and Methods

### 2.1. Data Sources

We initially selected nine *D. melanogaster* RNAseq ‘projects’ that each comprised at least 130 sequencing libraries, and downloaded them from the European Nucleotide Archive [42], Table 1). The selected datasets reflected a range of original experimental purposes. For example, an exploration of natural genetic variation in expression regulation in the Drosophila Genetic Reference Panel (DGRP) [43,44], the relationship between genotype and circadian gene expression [45], the utility of different bioinformatics pipelines [44], the impact of lead exposure on expression across development [46], and the role of selection in sex-biased gene expression [47]. Two projects lacked any evidence of viral infection (PRJNA527284 and PRJNA52728), and these were excluded. Two projects comprised a mix of developmental stages and/or cell culture (PRJNA305983 and PRJNA75285), and from these, we retained only adult data.

### 2.2. Virus Detection and Quantification of Host Expression

To identify unknown and potentially novel viral infections, we de novo assembled non-fly reads. We first excluded any reads mapping to *D. melanogaster* or to well-studied contaminating cellular organisms such as bacteria, fungi, trypanosomatids, and microsporidia (a ‘hologenome’ as reported in [58]) using Bowtie 2 [59]. We then assembled the remaining read pairs using Trinity [60], and retained all scaffolds with a length of at least 500 nt. We grouped the resulting scaffolds into clusters meeting at least a 95% sequence identity threshold using CD-HIT [61]. Cluster representatives were then used to search against a custom database using Diamond blastx [62], retaining clusters with a score at most 5% lower than the best alignment score for each query. This custom target database comprised all viral proteins from the Genbank non-redundant (nr) protein database [63] and all the prokaryote, protist, fungal, nematode, hymenopteran, and dipteran sequences from NCBI refseq protein database [64]. Contigs from each virus were then assembled together using Geneious Prime. These manually curated viral references were then used as targets for viral quantification by mapping using Bowtie 2 [59]. To mitigate the potential impact of barcode-switching [65], each virus was considered to be present in a library if the number of reads from that virus was at least 1% of the number in the library with the highest number of reads from that virus. We additionally applied a minimum threshold of 150 reads, chosen as this threshold reduced the inconsistency between duplicate samples (Figure S1). For comparison, we also estimated virus presence/absence in each library by estimating the rate of barcode switching based on sex-specific genes, but this approach gave similar results (Document S1).

To quantify fly gene expression, we mapped reads as pairs to the *D. melanogaster* genome (FlyBase release r6.27, FEB2019, [66]) using the splice-aware mapper, STAR [67] and counted the mapped reads using ‘featureCounts’ [68]. We verified the reported sex of each fly by counting reads mapping to twelve male-specific and five female-specific genes (FBgn0011669, FBgn0011694, FBgn0046294, FBgn0053340, FBgn0250832, FBgn0259795, FBgn0259971, FBgn0259975, FBgn0262099, FBgn0262623, and FBgn0270925 versus FBgn0000355, FBgn0000356, FBgn0000358, FBgn0000359, FBgn0000360, respectively). All gene counts from featureCounts and metadata including the number of reads that mapped to each virus per dataset are presented in (Zip File S1).

### 2.3. Association between Coinfecting Viruses in Each Dataset

To test if there is an interaction between co-infecting viruses, we generated a contingency table of the total number of infected and uninfected libraries for each pair of viruses in each dataset. We then tested for statistical significance of association between the viruses using Fisher’s exact test in the R stats package [69].

### 2.4. Virus Prevalence and Diversity

We quantified virus prevalence as the proportion of sequencing libraries within each published project for which the virus read number passed our presence/absence threshold (selected based on the inferred barcode switching rate, above). We then assumed binomial sampling to obtain confidence intervals for the proportion of affected libraries in each project. To explore the phylogenetic relationships between laboratory viruses and previously published virus sequences, we selected the sample within each project that had the highest virus read number for each virus. For galbut virus, which has two distinct strains, we selected the sample with the highest read number for each haplotype. We then performed phylogenetic analyses using the sequences from these samples and incorporating all the other sequences from each virus available in GenBank [63]. For analysis of segmented viruses, we selected the segment that had the most examples in genbank. The number of sequences available varied between 3 (Brandeis virus) and 144 (DMelSV), and the aligned sequence length varied between 1.4 kb (galbut virus) and 11.3 kb (nora virus). DNA sequences were aligned using MAFFT [70], and we inferred maximum likelihood trees using iqtree version 2 [71], using the best supported substitution model according to the Bayesian information criterion. Note that recombination is common in many of these viruses [29], such that trees should be interpreted in terms of overall similarity rather than relationship, and that branch lengths will not be proportional to divergence time. All alignments, model details, and trees are presented in (Zip File S2).

### 2.5. Differential Host Gene Expression

We performed a differential gene expression analyses using ‘Dream’ [72], an R package [69] for gene expression analysis in R that permits the use of mixed effect models. Each virus was analysed separately by combining all the project datasets in which it was detected, treating the presence/absence of each virus in each library as a fixed effect in the differential expression model. Host sex was also fitted as a fixed effect, as was its interaction with the presence/absence of the virus. The genotype and dataset project codes were combined and fitted as a random effect to account for differences between genetic background and laboratory environment. If different treatment methods such as lead exposure, mating statuses, or tissues were reported, these were also combined and included in the random effect. If both sexes were present, uninfected females were treated as the reference condition for comparisons. For example, if two viruses were present in a study, and both sexes were sampled from multiple genotypes and tissues, our analysis model would be:

Expression ~ Sex + Virus + Sex:Virus + (1 | Dataset_Genotype_Tissue)

If the virus was present in only one sex in a mixed-sex project dataset, or the infected project datasets had only one sex, then the sex term and its interaction with virus were excluded. If a virus was present in only one dataset, then the dataset term was excluded from the model, and if the dataset had only one genotype, then the genotype term was also removed accordingly. Fly age was not incorporated into the models because these datasets are from published studies that do not report fly age. Cut-off log-fold changes, logFC > 0.5 and logFC < −0.5, were used to define increased and decreased expression, respectively, and a Benjamini–Hochberg adjusted *p*-value < 0.001 (to account for multiple testing) was used to indicate statistical significance. The genes with significant changes in expression from the differential expression analysis were subsequently analysed for gene ontology enrichment using VISEAGO [73], which depends on TOPGO [74].

### 2.6. Correlation Analysis

To compare the effects on the host by viral infection post hoc, we calculated a Pearson correlation matrix between the estimated changes in gene expression in response to viruses and their interactions with sex using the rcorr function in the Hmisc package [75] in R [69].

## 3. Results

### 3.1. Ten RNA Viruses Were Detectable in Laboratory RNAseq Datasets

We examined a total of nine publicly available RNAseq projects, but two of these contained no viral reads and were excluded from further analyses (Table 1). One project (PRJNA258012) contained reads from Flock House virus, Newfield virus, Drosophila Totivirus, and Drosophila X Virus (DXV). All of these viruses are common cell culture contaminants [29], and three have only previously been reported from cell culture. In addition, read numbers were very highly correlated among these viruses across sequencing libraries (r ≥ 0.96, *p*-value = 2.2 × 10^−16^), consistent with a common source (Figure S2). We therefore chose to exclude these viruses from further analysis, as we believe they are likely to represent RNA contamination or barcode hopping from unreported cell culture libraries, rather than infections of the sequenced flies. Neither of the remaining two viruses seen in this project have been reported from cell culture. After excluding these putatively contaminating reads, we detected a total of 10 viruses with a high level of confidence, i.e., at high copy number in multiple libraries. These included galbut virus (*Partitiviridae*), Motts Mill virus (*Solemoviridae*), DAV (*Permutotetraviridae*), DCV *(Dicistroviridae),* nora virus (unclassified *Picornovirales*), Dmel sigma virus (*Rhabdoviridae*), Bloomfield virus (*Reoviridae*), Craigie’s Hill virus (*Nodaviridae*), Thika virus (unclassified *Picornavirales*), and Brandeis virus (cf. *Negevirus*). Out of a total of 2586 libraries across the seven datasets analysed, 625 (24%) were infected with only one virus and 104 (4%) were infected with two or more viruses. Using a *p*-value < 0.01 as a test of significance, we found that co-infecting viruses were independent of each other in the datasets used in this study, except for galbut virus and Motts Mill virus in PRJNA258012 (*p*-value = 3.4 × 10^−5^) and PRJNA281652 (*p*-value = 3.2 × 10^−5^), DAV and Thika virus in PRJNA261333 (*p*-value = 5 × 10^−3^), DAV and DCV in PRJNA281652 (*p*-value = 5 × 10^−4^), and DAV and nora virus in PRJNA518903 (*p*-value = 4 × 10^−4^). However, the number of coinfections was small; for example, in PRJNA258012, 49 (6.8%), and 171 (23.9%) libraries were infected with Motts Mill virus and galbut virus, respectively whilst one (0.1%) library was infected with both viruses. Similarly, in PRJNA281652, 45 (9.8%) and 79 (17.2%) libraires were infected with DCV and DAV, respectively whilst 17 (3.7%) libraries were infected with both viruses. In PRJNA518903, 10 (1.3%) and 31 (4%) libraires were infected with DAV and nora virus, respectively whilst four (0.5%) libraries were infected with both viruses. Finally, in PRJNA261333, 24 (6.1%) and 39 (9.8%) libraires were infected with DAV and Thika virus, respectively whilst seven (1.8%) libraries were infected with both viruses. Thus, the linear model should successfully disentangle the effects of the viruses, and any (unmodelled) interaction between viruses would only add to the residual variance.

Some samples displayed an extremely high virus titre (Zip File S1). Most extreme was library SRR3654766 (heads from lead-treated males), in which ca. 70% of non-ribosomal RNA reads derived from DCV. Additionally, at an occasionally very high level were nora virus and DAV, which constituted 46% of non-ribosomal reads in SRR8522465 and 25% in SRR8522473, respectively (adult guts from DGRP lines 309 and 359). The other viruses generally had a much lower copy-number, with Motts Mill virus reaching a maximum of 4.8% of non-ribosomal RNA reads (SRR7620105; adult males from DGRP line 93), Brandeis virus 3.8% (SRR3654657; heads from adult males), Thika virus 2.3% (SRR1577470; adult male Df(2L)ED247/+ flies), and Bloomfield virus 2.4% (SRR8522432; adult guts from DGRP line 380).

**Figure 1:**
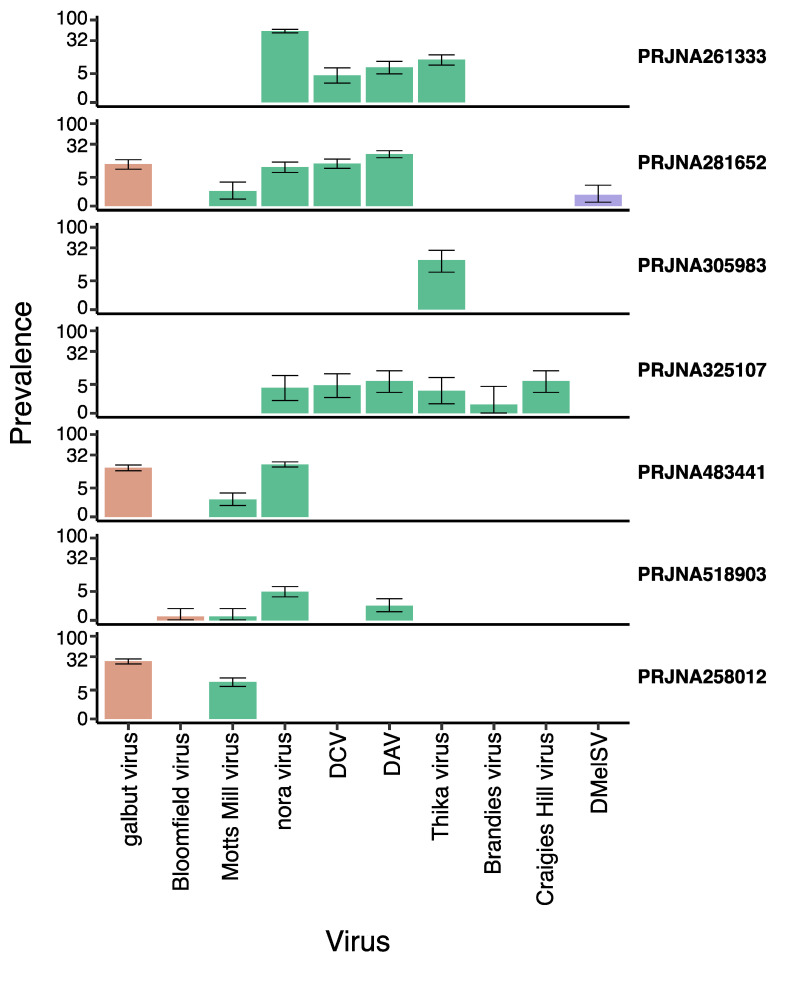
Prevalence of Viruses in the laboratory *D. melanogaster*. The figure shows the prevalence of galbut virus, Motts Mill virus, DAV, DCV, nora virus, DmelSV, Bloomfield virus, Craigie’s Hill virus, Thika virus, and Brandeis virus in the seven projects analysed. The dataset project codes are shown for each, and brown, green, and mauve bar charts represent dsRNA, +ssRNA, and −ssRNA viruses. Prevalence is given as a percentage of libraries in which the virus was detected, plotted on a log scaled axis for clarity, with 95% confidence intervals assuming binomial sampling.

Of these 10 viruses, nora virus had the highest prevalence, detectable in five projects, with a prevalence of up to 53% (PRJNA261333; Figure 1). In PRJNA518903, 100% of the nora-virus-infected libraries were gut tissue—consistent with its known gut tropism [76]. DAV had the second highest prevalence, occurring in four projects with a prevalence of up to 17% (PRJNA281652). The rarest viruses were Craigie’s Hill virus, Bloomfield virus, DMelSV and Brandeis virus, each appearing in one project at prevalences as low as 0.3% (Bloomfield virus in PRJNA518903; Figure 1). The two viruses that are thought to be exclusively vertically transmitted (DMelSV and galbut virus; [35,77] were only found in DGRP lines, consistent with those lines’ relatively recent wild origin. And, consistent with previous studies of galbut virus [29], we detected two distinct strains with pairwise sequence identity of 85% in DGRP projects PRJNA258012 and PRJNA483441. It is noteworthy that no laboratory isolates have been published for Thika virus, Motts Mill virus, Brandeis virus, Craigie’s Hill virus, and Bloomfield virus, potentially allowing us to analyse *Drosophila* expression in response to these pathogens for the first time.

### 3.2. Laboratory Viruses Are Closely Related to Each Other

Our phylogenetic analyses showed that although laboratory viruses were very rarely identical across projects, viruses from different projects sometimes clustered together with each other and previously published laboratory isolates. This was most notable for DAV, DCV, and Nora virus (Figure S3A–C), and may suggest that there are clades of these viruses circulating in the laboratory environment. The same may be true for Motts Mill virus, as the four sequences formed two near-identical pairs, but previous laboratory isolates have not been reported (Document S2).

### 3.3. Many Laboratory Virus Infections Did Not Induce Detectable Changes in Gene Expression

**Figure 2:**
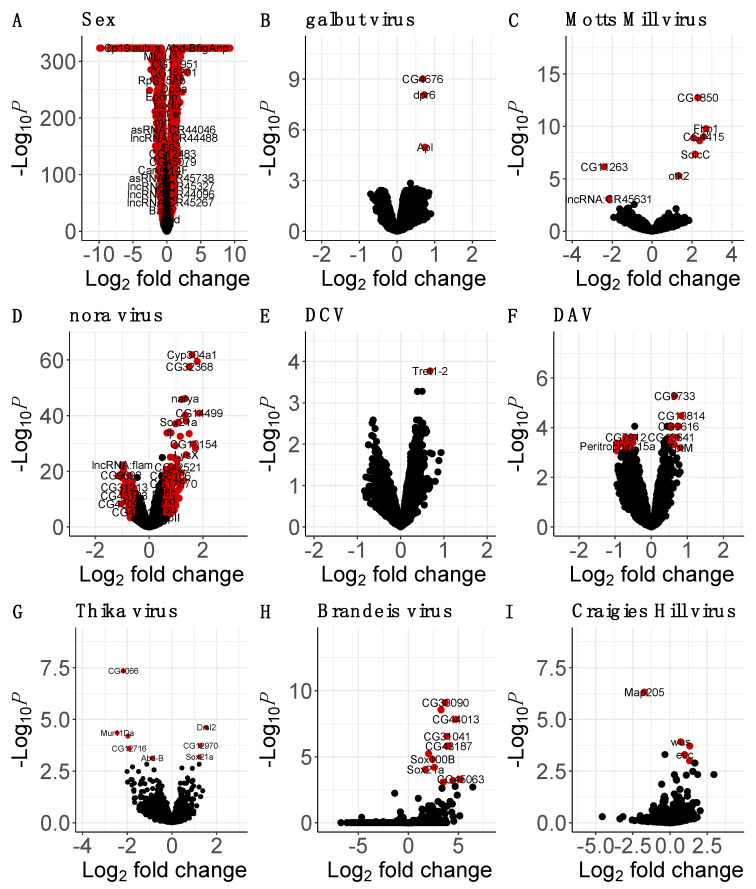
Effects of viral infection on host gene expression. Using uninfected females as baseline, the volcano plots show the main effects on *Drosophila* gene expression of sex (i.e., being male) in panel (**A**), and viral infection in panels (**B**–**I**). The virus names and total number of variables (genes) used in each expression analysis is shown. A significance threshold of *p* < 0.001 was used and a logFC cutoff of 0.5. The genes in red are both nominally significant and have a 0.5 < logFC < −0.5.

We did not detect any significant changes in gene expression in response to DMelSV (Spreadsheet S1; Figure S4), and most of the expression changes in response to Bloomfield virus (Figure S4), galbut virus, and DCV were similarly not significant (Figure 2). *Tret1-2*, which encodes a sugar transporter [78], was the only gene with a significant change in expression in response to DCV, and *dpr6*, *CG4676*, and *Apl* were the only genes with significant increase in response to galbut virus infection. *Defective proboscis extension response 6* (*dpr6*) is involved in synapse organisation [79] whilst *CG4676* and *Apl* are involved in protein transport and localisation [80,81]. In Bloomfield-virus-infected flies, only three genes, *Ets21C*, *CG16995*, and *CG18649*, were significantly upregulated (Figure S4). *Ets at 21C* is involved in response to stress such as infection or oncogene activation, *CG16995* is involved in sexual reproduction, while the function of *CG18649* is still unknown.

### 3.4. Motts Mill Virus and Craigies Hill Virus Affected the Expression of Genes Related to Development

Of the 454 genes that displayed a logFC of more than 0.5 in response to Craigies Hill virus infection, only 4 were nominally significant (adjusted *p*-value threshold of 0.001; Figure 2). The significantly upregulated genes in response to Craigies Hill virus infection included *esc* and *wus*, which are involved in development [82,83], and *CG31704* that is involved in sexual reproduction [84]. *Microtubule-associated protein 205* (*Map-205*) that is involved in mitosis [85], was the only significantly downregulated gene in response to Craigies Hill virus infection.

Of the 1865 genes that showed a logFC of more than 0.5 in response to Motts Mill virus infection, only 7 were nominally significant (adjusted *p*-value threshold of 0.001; Figure 2). The upregulated genes in response to Motts Mill virus infection include *Lsp1alpha* and *Fbp1* that encode macromolecular components [86,87], *SdicC* that is involved in microtubule transport, and *tipE* that enhances para sodium ion channel function and is required during pupal development to rescue adult paralysis [88]. Of the 3275 genes that showed displayed a logFC of less than 0.5 in response to Motts Mill virus infection, only 2 were nominally significant (adjusted *p*-value threshold of 0.001; Figure 2). These two downregulated genes were lncRNA:CR45631 and CG11263, which encode a long, non-coding RNA and an RNA binding protein.

### 3.5. Brandeis Virus and Thika Virus Affected the Expression of Genes Related to Reproduction and Immunity

Out of 1082 genes that showed a logFC of more than 0.5 in response to Brandeis virus, 14 were nominally significant (adjusted *p*-value threshold of 0.001; Figure 2). These included *CG31704* and *CG3349*, which are involved in male sexual reproduction [84,89]. Other significantly upregulated genes in response to Brandeis virus include *SPH93* and *Ir56b*, which take part in host antimicrobial defence [90] and the response to chemical stimuli, respectively [91]. There were no significantly downregulated genes in response to Brandeis virus.

Out of 240 genes that showed a logFC of more than 0.5 in response to Thika virus infection, 3 were nominally significant (*p*-value threshold of 0.001; Figure 2). These were *CG12970*, which is involved in the STING antiviral response [3] and *Drsl2*, which encodes a peptide with homology to the antifungal peptide encoded by *Drs* [92]. Another gene, *Sox21a*, which is involved in stem cell differentiation in the midgut, was also significantly upregulated in response to Thika virus infection [93]. Out of the 394 genes that displayed a logFC of more than −0.5 (adjusted *p*-value threshold of 0.001; Figure 2) in response to Thika virus infection, 5 were nominally significant. Most of the significantly downregulated genes in response to Thika virus infection are involved in sexual reproduction. These included genes encoding chorion proteins such as *CG4066*, *CG12716*, and *Mur11Da* [94], and *Abd-B*, which is involved in regulating post-mating responses in females [95].

### 3.6. The Enteric Viruses Nora Virus and DAV May Trigger Host Innate Immune Response and Gut Epithelium Repair

Of the 340 genes that showed a logFC of more than 0.5 in response to nora virus infection, 330 were significant (adjusted *p*-value threshold of 0.001; Figure 2). Many of the significantly upregulated genes in response to nora virus infection are involved in midgut stem cell differentiation. These include *Ser12*, which is predicted to encode a protein implicated in wound healing [96], and *Ptx1*, *Sox21a*, and *GATAe*, which encode proteins implicated in differentiation in the midgut stem cell [93,97,98]. *Unpaired3* (*upd3*), which is involved in gut epithelium repair via the JAK/STAT pathway was also significantly upregulated in response to nora virus infection. Numerous immune response genes were significantly upregulated in response to nora virus infection. These include *Nazo*, which encodes an antiviral effector protein that is expressed downstream of Sting and relish antiviral response [99], *DptA* and *DptB*, which both encode antimicrobial peptides regulated by the ImD pathway [100,101], and *Mtk*, which encodes an antifungal peptide regulated by the ImD and Toll pathways [102].

Of the 314 genes that showed a logFC of less than −0.5 in response to nora virus infection, 313 were significant (adjusted *p*-value threshold of 0.001; Figure 2). These significantly downregulated genes include numerous genes that encode structural constituent of the chitin-based larval cuticle such as *Lcp65Ac*, *TwdlR*, and *TwdlS* [103,104,105]. There was also a downregulation of genes involved in mitochondrial function such as *Mics1* and *fzo*, which encode proteins that enhance oxidative phosphorylation and enable the fusion of the mitochondrion during spermatid differentiation, respectively [106,107].

Of the 43 genes that showed a logFC of more than 0.5 in response to DAV, 12 were significant (adjusted p-value threshold of 0.001; Figure 2). Antimicrobial genes such as *Srg1*, *CG6429*, and *TotM* were significantly upregulated in response to DAV infection. *Srg1* is involved in STING antiviral signalling, while *CG6429* and *TotM* are predicted to be involved in immune response [108,109]. Of the 397 genes that showed a logFC decrease of more than −0.5 in response to DAV infection, 25 were significant (adjusted *p*-value threshold of 0.001; Figure 2). Most of the significantly downregulated genes in response to DAV were long, non-coding RNA such as *lncRNA:CR45910* and *lncRNA:CR44953*, with unknown function. In response to DAV, many genes predicted to be involved in fatty acid-CoA metabolism such as *CG31091* [110], *CG6300* [111], and *Traf-like* [112] were significantly downregulated. We also observed a significant downregulation of *Peritrophin-15a* and *E(spl)malpha-BFM*, which are involved in chitin binding and development [113] and cell fate specification and sensory organ development via Notch signaling [114].

### 3.7. Sex–Virus Interaction

**Figure 3:**
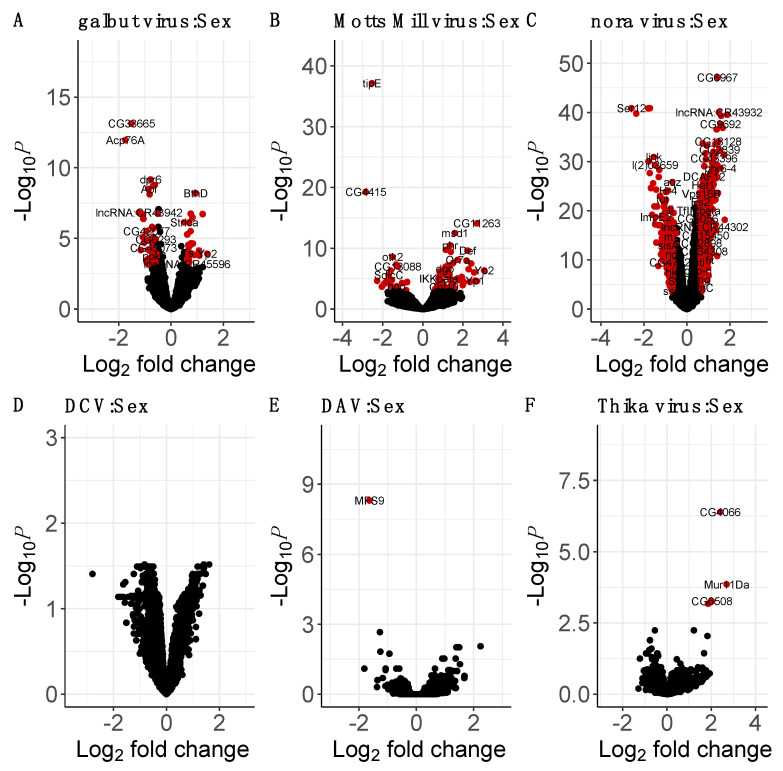
Sex–virus interaction effects on host gene expression. Using uninfected females as baseline, the volcano plots show the sex interaction with viral infection (**A**–**F**), that is, the difference in expression between virus-infected males and the expression that would be predicted from the combined effects of sex and infection alone. The virus names and total number of variables (genes) used in each expression analysis are shown. A significance threshold of *p* < 0.001 was used as well as a logFC cutoff of 0.5. The genes in red are both significant and have a 0.5 < logFC < −0.5.

The interaction between virus and sex could only be inferred for viruses that were present in both sexes, namely galbut virus, Motts Mill virus, nora virus, DCV, DAV and Thika virus (Spreadsheet S2; Figure 3). The effect of sex on the host response to DCV was not significant for any genes in our analysis. However, in the flies infected with DAV, Thika virus, Motts Mill virus, galbut virus, and nora virus, the effect of sex on virus infection induced significant changes in the expression of 1, 4, 83, 95, and 1504 genes, respectively (adjusted *p*-value threshold of 0.001; Figure 3). Only *MFS9*, which is predicted to be involved in transmembrane anion transport [115], was significantly downregulated in males infected with DAV. Four genes, *Mur11Da*, *CG4066*, *CG31661*, and *CG6508,* were significantly upregulated in Thika-virus-infected males. *Mucin related 11Da*, *CG4066,* and *CG31661* are predicted to be involved in chorion eggshell assembly [94] and *CG6508* is predicted to enable endopeptidase activity [116].

In galbut-virus-infected males, there was a significant upregulation of numerous genes involved in cuticle development such as *Lcp4* and *Cpr65Ec* [104] and immune response such as *AttC,* which shows homology to several antimicrobial peptides [117]. In galbut-virus-infected males, there was also a significant downregulation of male reproductive genes such as *Sfp24Ba* and *Acp76A* [84], metal ion transport genes such as *dpr3* [118], and several long, non-coding RNA genes (Figure 3). In Motts-Mill-virus-infected males, there was a significant upregulation of genes involved in the JAK/STAT pathway, such as *GILT2* and *GILT3* [119]; and the Imd pathway, such as *Def* and *IKKbeta* ([120,121]; Figure 3). There was also a significant upregulation in the expression of *tipE*, which encodes a protein that enhances sodium ion channel function [88] and a significant downregulation of genes implicated in development such as *otk2* and *lov* in Motts-Mill-virus-infected males [122,123].

In nora-virus-infected males, there was a significant upregulation of genes such as *Mur82C* and *Cpr56F*, which are predicted to encode structural components of extracellular matrix ([124,125]; Figure 3). There was also a significant upregulation of genes involved in systemic immune response such as such as *CecA1* and *Def*, which encode an antimicrobial peptide regulated by the ImD and Toll pathways [120] and male reproductive genes such as *Sfp84E* and *Sfp33A2* [84,126]. Nora-virus-infected males displayed a significant downregulation of immune genes such as *Ser12*, which is involved in wound healing [127] and *Send2*, which encodes a protein stored in the seminal receptacle and which is predicted to be involved in proteolysis [128]. The genes, *narya* and *ImpE2*, which are involved in DNA repair and embryogenesis, respectively, were also significantly downregulated in males infected with Motts Mill virus [129,130].

### 3.8. Changes in Gene Expression Were Positively Correlated between Flies Infected with Galbut Virus, Nora Virus, and Motts Mill Virus

**Figure 4:**
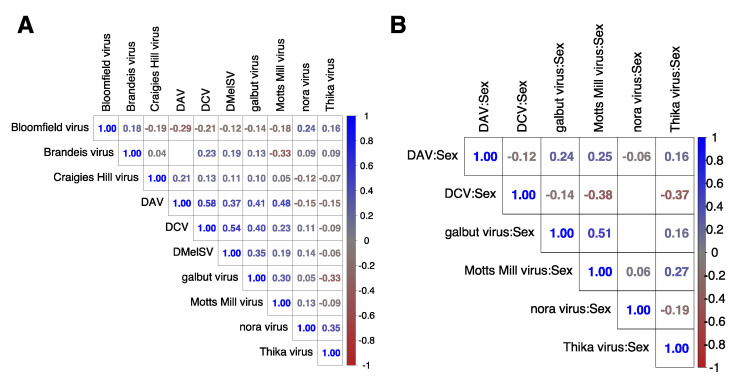
Correlation. The triangular matrix shows significant (*p* < 0.001) Spearman rank correlation coefficients between the estimated changes in gene expression induced by 10 *Drosophila* viruses. The virus names are shown above and to the side. Panel (**A**) shows the correlation between changes in expression in response to viruses. Panel (**B**) shows the correlations between the changes in expression induced by the effects of sex on viral infection. Blank squares have non-significant correlations.

To determine whether changes in gene expression in response to virus challenge were consistent across viruses, we compared the inferred changes between each of the viruses (Figure 4). The magnitude of such correlations was generally small, with the exception of the expression changes induced by galbut virus, DAV, and Motts Mill virus. Changes in gene expression were significantly positively correlated (r = 0.53, *p*-value < 0.001) between the DAV, galbut virus, and Motts Mill infection, although none were significant in all three (Figure 4). Changes in gene expression were significantly positively correlated (r = 0.53, *p*-value < 0.001) between the galbut:sex and Motts Mill virus:sex. That is, the effect of sex on virus infection induced similar changes in the expression of 209 genes in galbut-virus- and Motts-Mill-virus-infected flies, with four genes being nominally significant in both infections. These genes include three long, non-coding RNA, lncRNA:CR40465, lncRNA:CR44631, lncRNA:CR44289, and *Yp2*, which encodes the major yolk protein of eggs.

### 3.9. Significantly Enriched GO Terms

**Figure 5:**
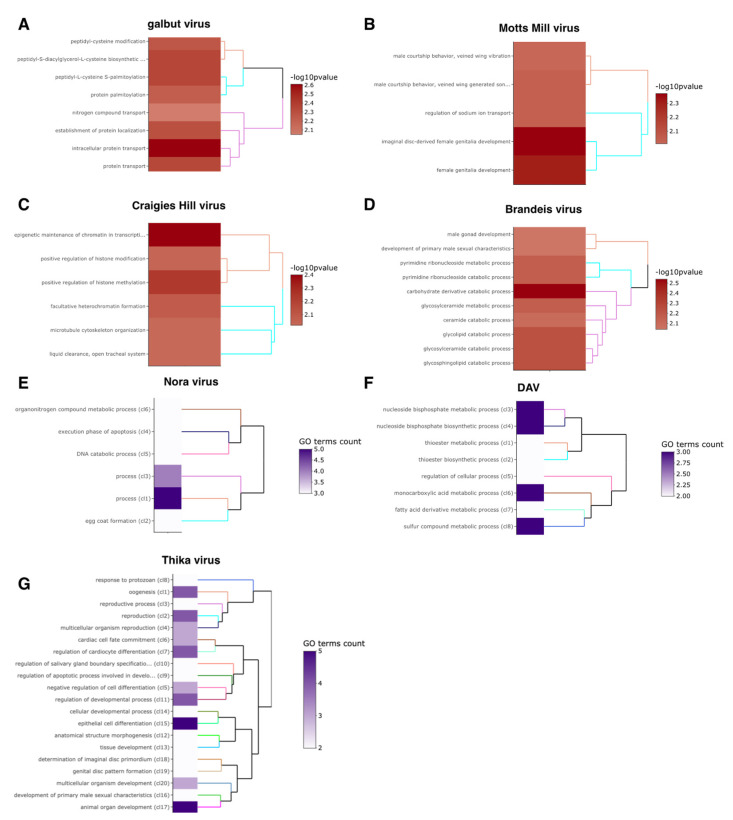
Main effects significantly enriched GO terms. GO enrichment on the effect of virus infection on *Drosophila* gene expression. The virus names are shown. (**A**–**D**) show a −log10(*p*-value) of functional enrichment analysis and a dendrogram based on hierarchical clustering of the enriched GO terms. The heatmaps of functional GO terms show a clustering combining a description of the first common GO ancestor of each set of GO terms. The heatmap shows the number of GO terms in each set and the dendrogram is based on BMA semantic similarity distance and ward.D2 aggregation criterion (**E**–**G**).

**Figure 6:**
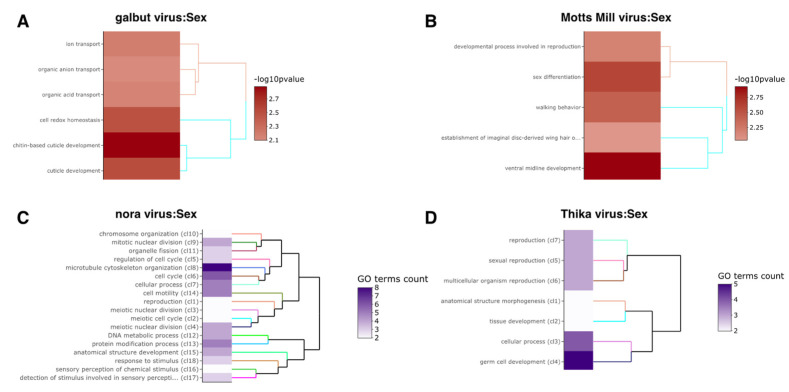
Sex–virus interaction significantly enriched GO terms. GO enrichment on the effect of sex on virus infection. The virus names are shown. (**A**,**B**) show a −log10(*p*-value) of functional enrichment analysis and a dendrogram based on hierarchical clustering of the enriched GO terms. (**C**,**D**) are heatmaps of functional GO terms clusters combining a description of the first common GO ancestor of each set of GO terms. The heatmap shows the number of GO terms in each set and the dendrogram is based on BMA semantic similarity distance and ward.D2 aggregation criterion.

There were no significantly enriched GO terms among the genes that changed expression in response to DmelSV, Bloomfield virus and DCV. Six GO terms related to cell proliferation were significantly enriched in Craigies-Hill-virus-infected flies (Spreadsheet S3; Figure 5). In general, the enriched GO terms were consistent with the genes that were differentially expressed in our analysis. For example, the significantly enriched terms in response to Thika virus and DAV infection include development and fatty acid metabolism, respectively (Figure 5). Similar consistencies between the significantly enriched GO terms and expression analysis were observed on the effect of sex on virus infection (Spreadsheet S4; Figure 6). For example, the effect of sex on Motts Mill virus and galbut virus infection significantly enriched GO terms such as ion transport, developmental process, and cuticle development (Figure 6). Similarly, the effect of sex on nora virus infection enriched GOP terms related to meiotic cell cycle and response to stimulus (Figure 6).

## 4. Discussion

Most studies of how *Drosophila* gene expression changes in response to virus infection either use non-native viruses or inject natural viruses into the host—which may not be a good reflection of what happens in nature. To understand how *Drosophila* gene expression changes in response to native virus infection, we analysed seven publicly available RNA-seq datasets that were serendipitously infected with ten different viruses.

### 4.1. Prevalence

The viruses found in this study were galbut virus, Motts Mill virus, DAV, DCV, nora virus, DmelSV, Bloomfield virus, Craigie’s Hill virus, Thika virus, and Brandeis virus. The most prevalent viruses in the datasets analysed were nora virus and DAV, with prevalences of up to 52% and 18%, respectively. This is higher than has been observed in wild flies; a study of wild *D. melanogaster* suggested that the average global prevalences of nora virus and DAV is about 7% each [29], although the authors also reported that DAV had a prevalence of about 50% in wild *D. melanogaster* collected from Athens (Georgia, USA). The prevalence of galbut virus in this study was 14%, which is much lower than has been reported in wild flies, where the prevalence of galbut is often over 50% [29,35]. The difference in virus prevalence between laboratory and wild flies indicates that some viruses may be better suited than others to the ecology of the lab, suggesting the need to isolate more natural viruses from wild *Drosophila* to better understand host–virus interactions.

### 4.2. Changes in Gene Expression in Virus-Infected Flies

In response to DAV and nora virus infection, we found evidence of increased expression of immune genes involved in the antiviral STING pathway and changes in the expression of genes involved in lipid metabolism. Recent work has shown that flies mutant in STING display reduced lipid storage and downregulated expression of lipid metabolism genes [131]. The authors also reported that *Drosophila* STING interacts with lipid-synthesizing enzymes such as acetyl-CoA carboxylase [131]. It has also been reported that genes regulated by IKKβ, such as STING, restrict infection by picorna-like viruses in *Drosophila* S2 cells [132], which is consistent with our findings given that nora virus is a picorna-like virus.

As in previous studies, we also detected a significant downregulation of genes involved in mitochondrial function in response to nora virus infection. For example, using DNA microarray, [12] reported a significant downregulation of *CG15434* that is predicted to be involved in mitochondrial electron transport. The authors also found significant upregulation of chorion protein genes [12] such as *Femcoat* and *Cp18*, which were not differentially expressed in our study. In contrast to our findings, a study on *Drosophila*’s response to nora virus infection reported a downregulation of antimicrobial peptide genes regulated by the IMD pathway, such as *DptA* [133], which were upregulated in our study. The authors also observed a significant upregulation of immune response genes such as *Tep4* and *Drs* [133], which were not differentially expressed in our study.

Although we detected no significant changes in expression in response to DMelSV, it has been reported that DMelSV infection increases the rates of expression of chorion protein genes such as *Cp18* and downregulation of ribosomal protein genes [134]. The authors also reported no differentially expressed immune genes in response to DMelSV [134]. However, another study found an increase in DMelSV replication in *PGRP-LC* and *domeless* knockout flies, suggesting the recruitment of IMD and JAK/STAT pathways respectively in DMelSV infection [135].

In our study, only the gene encoding a trehalose transporter, *Tret1-2*, was significantly upregulated in response to DCV infection. This is in contrast with published work that showed that systemic DCV infection decreases the expression of many genes involved in trehalose metabolism, such as *Tret1-1*, *CG5177*, and *Tps1* [136]. Using Drosophila S2 cells, another study reported that only a few genes were differentially expressed in response to DCV infection, with many of the upregulated genes encoding for heat shock proteins [13]. DCV infection has also been found to increase the expression genes immune genes such as *Spz* [9] and *vir-1* [10] that are involved in the toll and JAK/STAT pathways, respectively.

### 4.3. Potential Virus-Induced Pathologies

The changes in expression we have observed may be indicative of virus-induced pathologies in the host. For example, GO analysis showed that changes in the expression of metal ion transport was significantly enriched in the flies infected with Motts Mill virus. Perhaps, virus infection may have induced the deregulation of metal ion transport in flies infected with Motts Mill, leading to their change in expression. Conversely, there was an increase in the expression of *tipE* in response to Motts Mill infection, which may have induced changes in the function of the metal ion channels. *Temperature-induced paralytic E* (*tipE*) encodes a protein with a cysteine-stabilized αβ scaffold (CSαβ). *Drosophila* peptides with CSαβ scaffold are implicated in the regulation of voltage-gated sodium ion channels [137].

We observed an increase in the expression of genes involved in chitin biogenesis and binding, wound healing, and differentiation in the midgut stem cell, respectively. Perhaps, in response to nora-virus-induced damage of the gut epithelium [76], the host may activate the JAK/STAT pathway via upd3 in the gut to regulate the repair response. Published work has shown that upd genes are expressed by haemocytes upon gut septic injury, to remotely stimulate stem cell proliferation and the expression of *Drosomycin*-like genes in the intestine [138]. This is consistent with work showing that *upd3* and the chitin metabolism gene, *CG32302*, were significantly upregulated in *Drosophila* infected with nora virus [12,133]. Genes involved in midgut stem cell differentiation such as *Sox21a* were also upregulated in response Thika virus infection in our study, which is in contrast with published work. For example, differential gene expression analysis on *Drosophila* midgut cells detected a significant downregulation of *Socs36E*, which is upregulated following gut septic injury [138] and encodes a negative regulator of the JAK/STAT pathway [139]. Although not detected in our study, it has been reported that systemic DCV infection induces expression of genes consistent with nutritional stress in the digestive tract [136]. The authors found that many of the repressed genes in response to DCV infection are also downregulated in flies undergoing starvation, which include the protease gene, *Jon65Ai*, and the lysozyme gene, *LysE* [136].

Overall, our work confirms that viruses are often naturally present in laboratory *Drosophila* fly culture, and that they can induce detectable changes in host gene expression. It may therefore be useful to consider their effects in experimental studies, especially those that generate transcriptomic data.

## Figures and Tables

**Table 1:** Datasets examined.

Project Code	Number of Libraries	Data	Genotype	Sex	Number of Reads	Viruses Found	Reference
PRJNA258012	768	Single virgin adult flies	16 DGRP	Males and females	7.74 billion	Galbut virus, Motts Mill virus	[43,48]
PRJNA261333	396	Single, 3–5-day post-eclosion adult	99 DrosDel lines	Male and females	2.94 billion	DAV, DCV nora virus, Thika virus	[49,50]
PRJNA281652	919	Single virgin and mated adult fly heads	F1 of Female DGRP and Male w118 crosses	Females	2.27 billion	DAV, DCV, galbut virus, Motts Mill virus, nora virus, DmelSV	[51,52]
PRJNA305983	179	Single whole larvae, pupae & virgin adults	10 different *Drosophila* genotypes	Males and females	6.26 billion	Thika virus	[47,53]
PRJNA325107	158	Control and lead-exposed single adults	Adult w1118	Males	5.18 billion	Brandeis virus, Craigie’s Hill virus, DAV, DCV, Motts Mill virus, nora virus,	[46,54]
PRJNA483441	800	Pooled and mated adult lies	200 DGRP	Males and females	15.53 billion	Galbut virus, Motts Mill viurus, nora virus	[44]
PRJNA518903	778	Brain, abdominal fat body, whole gut without the crop, and malpighian tubes of mated flies	w1118 and DGRP	Males	1.54 billion	Bloomfield, DAV, Motts Mill, nora	[45]
PRJNA75285	699	Mixture of cell culture and adult flies	Not mentioned	Cell culture and Mixed-sex pools	22.94 billion	None	[55,56]
PRJNA527284	288	Single embryos	Fly lines (w1118)	N/A	894 million	None	[57]

## Data Availability

No new data were generated during this study. Analyses and scripts are available from https://github.com/okuyateh/Changes-in-gene-expression-due-to-natural-viral-infection-in-laboratory-Drosophila.

## Supplementary Materials

The following supporting information can be downloaded at https://www.mdpi.com/article/10.3390/v15091849/s1. Figure S1: Best threshold to estimate viral presence. Figure S2: Correlations between normalised reads in PRJNA258012. Figure S3: Phylogenies of nora virus, Thika virus, DAV, and DCV. Figure S4: Effects of viral infection on host gene expression. Document S1: Estimating barcode switching rates. Document S2: Phylogenies of virus sequences. Spreadsheet S1: Main effects expression data. Spreadsheet S2: Virus–sex interaction expression data. Spreadsheet S3: Main effects enriched GO terms. Spreadsheet S4: Virus–sex interaction enriched GO terms. Zip File S1: Metadata of datasets and count matrices from feature counts. Zip File S2: Virus sequence alignments, model details, and trees.

## References

[1] Mussabekova A., Daeffler L., Imler J.-L. (2017). Innate and intrinsic antiviral immunity in Drosophila. Cell Mol. Life Sci..

[2] Dostert C., Jouanguy E., Irving P., Troxler L., Galiana-Arnoux D., Hetru C., Hoffmann J.A., Imler J.-L. (2005). The Jak-STAT signaling pathway is required but not sufficient for the antiviral response of drosophila. Nat. Immunol..

[3] Holleufer A., Winther K.G., Gad H.H., Ai X., Chen Y., Li L., Wei Z., Deng H., Liu J., Frederiksen N.A. (2021). Two cGAS-like receptors induce antiviral immunity in Drosophila. Nature.

[4] Liu Q., Kausar S., Tang Y., Huang W., Tang B., Abbas M.N., Dai L. (2022). The Emerging Role of STING in Insect Innate Immune Responses and Pathogen Evasion Strategies. Front. Immunol..

[5] Palmer W.H., Joosten J., Overheul G.J., Jansen P.W., Vermeulen M., Obbard D.J., Van Rij R.P. (2019). Induction and Suppression of NF-κB Signalling by a DNA Virus of *Drosophila*. J. Virol..

[6] Shelly S., Lukinova N., Bambina S., Berman A., Cherry S. (2009). Autophagy Is an Essential Component of Drosophila Immunity against Vesicular Stomatitis Virus. Immunity.

[7] Wang X.-H., Aliyari R., Li W.-X., Li H.-W., Kim K., Carthew R., Atkinson P., Ding S.-W. (2006). RNA Interference Directs Innate Immunity Against Viruses in Adult *Drosophila*. Science.

[8] Zambon R.A., Nandakumar M., Vakharia V.N., Wu L.P. (2005). The Toll Pathway Is Important for an Antiviral Response in *Drosophila*. Proc. Natl. Acad. Sci. USA.

[9] Kemp C., Mueller S., Goto A., Barbier V., Paro S., Bonnay F., Dostert C., Troxler L., Hetru C., Meignin C. (2013). Broad RNA Interference–Mediated Antiviral Immunity and Virus-Specific Inducible Responses in *Drosophila*. J. Immunol..

[10] Hedges L.M., Johnson K.N. (2008). Induction of host defence responses by Drosophila C virus. J. Gen. Virol..

[11] Palmer W.H., Medd N.C., Beard P.M., Obbard D.J. (2018). Isolation of a natural DNA virus of Drosophila melanogaster, and characterisation of host resistance and immune responses. PLoS Pathog..

[12] Cordes E.J., Licking-Murray K.D., Carlson K.A. (2013). Differential gene expression related to Nora virus infection of Drosophila melanogaster. Virus Res..

[13] Merkling S.H., Overheul G.J., van Mierlo J.T., Arends D., Gilissen C., van Rij R.P. (2015). The heat shock response restricts virus infection in Drosophila. Sci. Rep..

[14] Zhu F., Ding H., Zhu B. (2013). Transcriptional profiling of Drosophila S2 cells in early response to Drosophila C virus. Virol. J..

[15] Gupta V., Vasanthakrishnan R.B., Siva-Jothy J., Monteith K.M., Brown S.P., Vale P.F. (2017). The route of infection determines *Wolbachia* antibacterial protection in *Drosophila*. Proc. R. Soc. B Boil. Sci..

[16] Mondotte J.A., Gausson V., Frangeul L., Blanc H., Lambrechts L., Saleh M.-C. (2018). Immune priming and clearance of orally acquired RNA viruses in Drosophila. Nat. Microbiol..

[17] Mondotte J.A., Saleh M.-C. (2018). Antiviral Immune Response and the Route of Infection in Drosophila melanogaster. Advances in Virus Research.

[18] Ferreira Á.G., Naylor H., Esteves S.S., Pais I.S., Martins N.E., Teixeira L. (2014). The Toll-Dorsal Pathway Is Required for Resistance to Viral Oral Infection in Drosophila. PLoS Pathog..

[19] Wong Z.S., Brownlie J.C., Johnson K.N. (2016). Impact of ERK activation on fly survival and Wolbachia-mediated protection during virus infection. J. Gen. Virol..

[20] Longdon B., Cao C., Martinez J., Jiggins F.M. (2013). Previous Exposure to an RNA Virus Does Not Protect against Subsequent Infection in Drosophila melanogaster. PLoS ONE.

[21] Tafesh-Edwards G., Eleftherianos I. (2020). Drosophila immunity against natural and nonnatural viral pathogens. Virology.

[22] van Mierlo J.T., Overheul G.J., Obadia B., van Cleef K.W.R., Webster C.L., Saleh M.-C., Obbard D.J., van Rij R.P. (2014). Novel Drosophila Viruses Encode Host-Specific Suppressors of RNAi. PLoS Pathog..

[23] Avadhanula V., Weasner B.P., Hardy G.G., Kumar J.P., Hardy R.W. (2009). A Novel System for the Launch of Alphavirus RNA Synthesis Reveals a Role for the Imd Pathway in Arthropod Antiviral Response. PLoS Pathog..

[24] Bronkhorst A.W., van Cleef K.W.R., Vodovar N., İnce İ.A., Blanc H., Vlak J.M., Saleh M.-C., van Rij R.P. (2012). The DNA virus Invertebrate iridescent virus 6 is a target of the *Drosophila* RNAi machinery. Proc. Natl. Acad. Sci. USA.

[25] Nayak A., Berry B., Tassetto M., Kunitomi M., Acevedo A., Deng C., Krutchinsky A., Gross J., Antoniewski C., Andino R. (2010). Cricket paralysis virus antagonizes Argonaute 2 to modulate antiviral defense in Drosophila. Nat. Struct. Mol. Biol..

[26] Medd N.C., Fellous S., Waldron F.M., Xuéreb A., Nakai M., Cross J.V., Obbard D.J. (2018). The virome of Drosophila suzukii, an invasive pest of soft fruit. Virus Evol..

[27] Shi M., White V.L., Schlub T., Eden J.-S., Hoffmann A.A., Holmes E.C. (2018). No detectable effect of *Wolbachia w* Mel on the prevalence and abundance of the RNA virome of *Drosophila melanogaster*. Proc. R. Soc. B Boil. Sci..

[28] Wallace M.A., Coffman K.A., Gilbert C., Ravindran S., Albery G.F., Abbott J., Argyridou E., Bellosta P., Betancourt A.J., Colinet H. (2021). The Discovery, Distribution, and Diversity of DNA Viruses Associated with *Drosophila Melanogaster* in Europe. Virus Evol..

[29] Webster C.L., Waldron F.M., Robertson S., Crowson D., Ferrari G., Quintana J.F., Brouqui J.-M., Bayne E.H., Longdon B., Buck A.H. (2015). The Discovery, Distribution, and Evolution of Viruses Associated with Drosophila melanogaster. PLoS Biol..

[30] Webster C.L., Longdon B., Lewis S.H., Obbard D.J. (2016). Twenty-Five New Viruses Associated with the Drosophilidae (Diptera). Evol. Bioinform..

[31] Habayeb M.S., Ekengren S.K., Hultmark D. (2006). Nora virus, a persistent virus in Drosophila, defines a new picorna-like virus family. J. Gen. Virol..

[32] Jousset F.X., Plus N., Croizier G., Thomas M. (1972). Existence in Drosophila of 2 groups of picornavirus with different biological and serological properties. Comptes Rendus Hebd. Des Seances L’Academie Des Sci. Ser. D Sci. Nat..

[33] Teninges D., Ohanessian A., Richard-Molard C., Contamine D. (1979). Isolation and Biological Properties of Drosophila X Virus. J. Gen. Virol..

[34] Bruner-Montero G., Luque C.M., Cesar C.S., Ding S.D., Day J.P., Jiggins F.M. (2023). Hunting Drosophila viruses from wild populations: A novel isolation approach and characterisation of viruses. PLoS Pathog..

[35] Cross S.T., Maertens B.L., Dunham T.J., Rodgers C.P., Brehm A.L., Miller M.R., Williams A.M., Foy B.D., Stenglein M.D. (2020). Partitiviruses Infecting Drosophila melanogaster and Aedes aegypti Exhibit Efficient Biparental Vertical Transmission. J. Virol..

[36] Echalier G. (1997). Drosophila Viruses and Other Infections of Cultured Cells. Drosophila Cells in Culture.

[37] Brosh O., Fabian D.K., Cogni R., Tolosana I., Day J.P., Olivieri F., Merckx M., Akilli N., Szkuta P., Jiggins F.M. (2022). A novel transposable element-mediated mechanism causes antiviral resistance in *Drosophila* through truncating the Veneno protein. Proc. Natl. Acad. Sci. USA.

[38] Gomariz-Zilber E., Thomas-Orillard M. (1993). Drosophila C virus and Drosophila hosts: A good association in various environments. J. Evol. Biol..

[39] Gupta V., Stewart C.O., Rund S.S.C., Monteith K., Vale P.F. (2017). Costs and benefits of sublethal Drosophila C virus infection. J. Evol. Biol..

[40] Brun G., Sigot A. (1955). Etude de la sensibilité héréditaire au gaz carbonique chez la Drosophile. II. —Installation du virus σ dans la lignée germinale à la suite d’une inoculation. Ann. Inst. Pasteur.

[41] Rogers A., Towery L., McCown A., Carlson K.A. (2020). Impaired Geotaxis as a Novel Phenotype of Nora Virus Infection of *Drosophila melanogaster*. Scientifica.

[42] (2022). European Nucleotide Archive. https://www.ebi.ac.uk/ena/browser/home.

[43] Lin Y., Golovnina K., Chen Z.-X., Lee H.N., Negron Y.L.S., Sultana H., Oliver B., Harbison S.T. (2016). Comparison of normalization and differential expression analyses using RNA-Seq data from 726 individual Drosophila melanogaster. BMC Genom..

[44] Everett L.J., Huang W., Zhou S., Carbone M.A., Lyman R.F., Arya G.H., Geisz M.S., Ma J., Morgante F., Armour G.S. (2020). Gene expression networks in the *Drosophila* Genetic Reference Panel. Genome Res..

[45] Litovchenko M., Meireles-Filho A.C.A., Frochaux M.V., Bevers R.P.J., Prunotto A., Anduaga A.M., Hollis B., Gardeux V., Braman V.S., Russeil J.M.C. (2021). Extensive tissue-specific expression variation and novel regulators underlying circadian behavior. Sci. Adv..

[46] Qu W., Gurdziel K., Pique-Regi R., Ruden D.M. (2017). Identification of Splicing Quantitative Trait Loci (sQTL) in Drosophila melanogaster with Developmental Lead (Pb^2+^) Exposure. Front. Genet..

[47] Ingleby F.C., Webster C.L., Pennell T.M., Flis I., Morrow E.H. (2016). Sex-biased gene expression in Drosophila melanogaster is constrained by ontogeny and genetic architecture. Genetics.

[48] Lin Y., Chen Z.-X., Oliver B., Harbison S.T. (2016). Microenvironmental Gene Expression Plasticity Among Individual *Drosophila melanogaster*. G3 Genes Genomes Genet..

[49] Lee H., Cho D.-Y., Whitworth C., Eisman R., Phelps M., Roote J., Kaufman T., Cook K., Russell S., Przytycka T. (2016). Effects of Gene Dose, Chromatin, and Network Topology on Expression in Drosophila melanogaster. PLoS Genet..

[50] Lee H., Cho D.-Y., Wojtowicz D., Harbison S.T., Russell S., Oliver B., Przytycka T.M. (2018). Dosage-Dependent Expression Variation Suppressed on the *Drosophila* Male *X* Chromosome. G3 Genes Genomes Genet..

[51] Fear J.M., León-Novelo L.G., Morse A.M., Gerken A.R., Van Lehmann K., Tower J., Nuzhdin S.V., McIntyre L.M. (2016). Buffering of Genetic Regulatory Networks in *Drosophila Melanogaster*. Genetics.

[52] Kurmangaliyev Y.Z., Ali S., Nuzhdin S.V. (2016). Genetic Determinants of RNA Editing Levels of ADAR Targets in Drosophila melanogaster. G3 Genes Genomes Genet..

[53] Djordjevic J., Dumas Z., Robinson-Rechavi M., Schwander T., Parker D.J. (2022). Dynamics of sex-biased gene expression during development in the stick insect *Timema californicum*. Heredity.

[54] Qu W., Gurdziel K., Pique-Regi R., Ruden D.M. (2018). Lead Modulates trans- and cis-Expression Quantitative Trait Loci (eQTLs) in Drosophila melanogaster Heads. Front. Genet..

[55] Kondo S., Vedanayagam J., Mohammed J., Eizadshenass S., Kan L., Pang N., Aradhya R., Siepel A., Steinhauer J., Lai E.C. (2017). New genes often acquire male-specific functions but rarely become essential in *Drosophila*. Genes Dev..

[56] Pasquier C., Robichon A. (2022). Temporal and sequential order of nonoverlapping gene networks unraveled in mated female Drosophila. Life Sci. Alliance.

[57] Liu J., Frochaux M., Gardeux V., Deplancke B., Robinson-Rechavi M. (2020). Inter-embryo gene expression variability recapitulates the hourglass pattern of evo-devo. BMC Biol..

[58] Wallace M.A. (2021). Virus Discovery, Dynamics, and Disease in a Wild Drosophila Community. Ph.D. Thesis.

[59] Langmead B., Salzberg S.L. (2012). Fast gapped-read alignment with Bowtie 2. Nat. Methods.

[60] Grabherr M.G., Haas B.J., Yassour M., Levin J.Z., Thompson D.A., Amit I., Adiconis X., Fan L., Raychowdhury R., Zeng Q.D. (2011). Full-length transcriptome assembly from RNA-Seq data without a reference genome. Nat. Biotechnol..

[61] Fu L., Niu B., Zhu Z., Wu S., Li W. (2012). CD-HIT: Accelerated for Clustering the next-Generation Sequencing Data. Bioinformatics.

[62] Buchfink B., Xie C., Huson D.H. (2014). Fast and sensitive protein alignment using DIAMOND. Nat. Methods.

[63] Clark K., Karsch-Mizrachi I., Lipman D.J., Ostell J., Sayers E.W. (2016). GenBank. Nucleic Acids Res..

[64] O’Leary N.A., Wright M.W., Brister J.R., Ciufo S., Haddad D., McVeigh R., Rajput B., Robbertse B., Smith-White B., Ako-Adjei D. (2016). Reference sequence (RefSeq) database at NCBI: Current status, taxonomic expansion, and functional annotation. Nucleic Acids Res..

[65] Griffiths J.A., Richard A.C., Bach K., Lun A.T.L., Marioni J.C. (2018). Detection and removal of barcode swapping in single-cell RNA-seq data. Nat. Commun..

[66] The Flybase Consortium (1996). FlyBase: The Drosophila database. Nucleic Acids Res..

[67] Dobin A., Davis C.A., Schlesinger F., Drenkow J., Zaleski C., Jha S., Batut P., Chaisson M., Gingeras T.R. (2013). STAR: Ultrafast universal RNA-seq aligner. Bioinformatics.

[68] Liao Y., Smyth G.K., Shi W. (2014). feature Counts: An efficient general purpose program for assigning sequence reads to genomic features. Bioinformatics.

[69] R Core Team (2021). R: A Language and Environment for Statistical Computing.

[70] Katoh K. (2002). MAFFT: A novel method for rapid multiple sequence alignment based on fast Fourier transform. Nucleic Acids Res..

[71] Minh B.Q., Schmidt H.A., Chernomor O., Schrempf D., Woodhams M.D., von Haeseler A., Lanfear R. (2020). IQ-TREE 2: New Models and Efficient Methods for Phylogenetic Inference in the Genomic Era. Mol. Biol. Evol..

[72] Hoffman G.E., Roussos P. (2021). Dream: Powerful differential expression analysis for repeated measures designs. Bioinformatics.

[73] Brionne A., Juanchich A., Hennequet-Antier C. (2019). ViSEAGO: A Bioconductor package for clustering biological functions using Gene Ontology and semantic similarity. BioData Min..

[B74-viruses-15-01849] 74.Alexa, A.; Rahnenfuhrer, J. TopGO: Enrichment Analysis for Gene Ontology, R Package Version 2.26.0; 2016.

[B75-viruses-15-01849] 75.Harrel, F.E., Jr. Hmisc: Harrell Miscellaneous 2022

[76] Habayeb M.S., Cantera R., Casanova G., Ekström J.-O., Albright S., Hultmark D. (2009). The Drosophila Nora virus is an enteric virus, transmitted via feces. J. Invertebr. Pathol..

[77] Contamine D. (2008). Sigma Rhabdoviruses. Encyclopedia of Virology.

[78] Kanamori Y., Saito A., Hagiwara-Komoda Y., Tanaka D., Mitsumasu K., Kikuta S., Watanabe M., Cornette R., Kikawada T., Okuda T. (2010). The trehalose transporter 1 gene sequence is conserved in insects and encodes proteins with different kinetic properties involved in trehalose import into peripheral tissues. Insect Biochem. Mol. Biol..

[79] Carrillo R.A., Özkan E., Menon K.P., Nagarkar-Jaiswal S., Lee P.-T., Jeon M., Birnbaum M.E., Bellen H.J., Garcia K.C., Zinn K. (2015). Control of Synaptic Connectivity by a Network of Drosophila IgSF Cell Surface Proteins. Cell.

[80] Bannan B.A., Van Etten J., Kohler J.A., Tsoi Y., Hansen N.M., Sigmon S., Fowler E., Buff H., Williams T.S., Ault J.G. (2008). The Drosophila protein palmitoylome: Characterizing palmitoyl-thioesterases and DHHC palmitoyl-transferases. Fly.

[81] Boeynaems S., Bogaert E., Michiels E., Gijselinck I., Sieben A., Jovičić A., De Baets G., Scheveneels W., Steyaert J., Cuijt I. (2016). Drosophila screen connects nuclear transport genes to DPR pathology in c9ALS/FTD. Sci. Rep..

[82] Lollies A., Krsmanovic T., Jussen L.C., Behr M. (2012). Wurst-Mediated Airway Clearance is Required for Postembryonic Development. J. Allergy Ther..

[83] Wang L., Jahren N., Vargas M.L., Andersen E.F., Benes J., Zhang J., Miller E.L., Jones R.S., Simon J.A. (2006). Alternative ESC and ESC-Like Subunits of a Polycomb Group Histone Methyltransferase Complex Are Differentially Deployed during *Drosophila* Development. Mol. Cell Biol..

[84] Findlay G.D., Yi X., MacCoss M.J., Swanson W.J. (2008). Proteomics Reveals Novel Drosophila Seminal Fluid Proteins Transferred at Mating. PLoS Biol..

[85] Dobbelaere J., Josué F., Suijkerbuijk S., Baum B., Tapon N., Raff J. (2008). A Genome-Wide RNAi Screen to Dissect Centriole Duplication and Centrosome Maturation in Drosophila. PLoS Biol..

[86] Burmester T., Antoniewski C., Lepesant J.-A. (1999). Ecdysone-regulation of synthesis and processing of fat body protein 1, the larval serum protein receptor of Drosophila melanogaster. JBIC J. Biol. Inorg. Chem..

[87] Wolfe J., Akam M.E., Roberts D.B. (1977). Biochemical and Immunological Studies on Larval Serum Protein 1, the Major Haemolymph Protein of Drosophila melanogaster Third-Instar Larvae. Eur. J. Biochem..

[88] Feng G., Deak P., Chopra M., Hall L.M. (1995). Cloning and functional analysis of tipE, a novel membrane protein that enhances drosophila para sodium channel function. Cell.

[89] Sitnik J.L., Gligorov D., Maeda R.K., Karch F., Wolfner M.F. (2016). The Female Post-Mating Response Requires Genes Expressed in the Secondary Cells of the Male Accessory Gland in *Drosophila melanogaster*. Genetics.

[90] Shukla A.K., Spurrier J., Kuzina I., Giniger E. (2019). Hyperactive Innate Immunity Causes Degeneration of Dopamine Neurons upon Altering Activity of Cdk5. Cell Rep..

[91] Dweck H.K., Talross G.J., Luo Y., Ebrahim S.A., Carlson J.R. (2022). Ir56b is an atypical ionotropic receptor that underlies appetitive salt response in Drosophila. Curr. Biol..

[92] Tian C., Gao B., Rodriguez M.d.C., Lanz-Mendoza H., Ma B., Zhu S. (2008). Gene expression, antiparasitic activity, and functional evolution of the drosomycin family. Mol. Immunol..

[93] Meng F.W., Biteau B. (2015). A Sox Transcription Factor Is a Critical Regulator of Adult Stem Cell Proliferation in the Drosophila Intestine. Cell Rep..

[94] Tootle T.L., Williams D., Hubb A., Frederick R., Spradling A. (2011). Drosophila Eggshell Production: Identification of New Genes and Coordination by Pxt. PLoS ONE.

[95] Gligorov D., Sitnik J.L., Maeda R.K., Wolfner M.F., Karch F. (2013). A Novel Function for the Hox Gene Abd-B in the Male Accessory Gland Regulates the Long-Term Female Post-Mating Response in Drosophila. PLoS Genet..

[96] Ross J., Jiang H., Kanost M.R., Wang Y. (2003). Serine proteases and their homologs in the Drosophila melanogaster genome: An initial analysis of sequence conservation and phylogenetic relationships. Gene.

[97] Okumura T., Takeda K., Kuchiki M., Akaishi M., Taniguchi K., Adachi-Yamada T. (2016). GATAe regulates intestinal stem cell maintenance and differentiation in Drosophila adult midgut. Dev. Biol..

[98] Vorbrüggen G., Constien R., Zilian O., A Wimmer E., Dowe G., Taubert H., Noll M., Jäckle H. (1997). Embryonic expression and characterization of a Ptx1 homolog in Drosophila. Mech. Dev..

[99] Zhang L., Xu W., Gao X., Li W., Qi S., Guo D., Ajayi O.E., Ding S.-W., Wu Q. (2020). lncRNA Sensing of a Viral Suppressor of RNAi Activates Non-canonical Innate Immune Signaling in Drosophila. Cell Host Microbe.

[100] Lee J.H., Cho K.S., Lee J., Yoo J., Lee J., Chung J. (2001). Diptericin-like protein: An immune response gene regulated by the anti-bacterial gene induction pathway in Drosophila. Gene.

[101] Lemaitre B., Reichhart J.-M., Hoffmann J.A. (1997). *Drosophila* host defense: Differential induction of antimicrobial peptide genes after infection by various classes of microorganisms. Proc. Natl. Acad. Sci. USA.

[102] Levashina E.A., Ohresser S., Bulet P., Reichhart J.-M., Hetru C., Hoffmann J.A. (1995). Metchnikowin, a Novel Immune-Inducible Proline-Rich Peptide from Drosophila with Antibacterial and Antifungal Properties. Eur. J. Biochem..

[103] Cornman R.S. (2009). Molecular Evolution of Drosophila Cuticular Protein Genes. PLoS ONE.

[104] Karouzou M.V., Spyropoulos Y., Iconomidou V.A., Cornman R., Hamodrakas S.J., Willis J.H. (2007). Drosophila cuticular proteins with the R&R Consensus: Annotation and classification with a new tool for discriminating RR-1 and RR-2 sequences. Insect Biochem. Mol. Biol..

[105] Zuber R., Wang Y., Gehring N., Bartoszewski S., Moussian B. (2020). Tweedle proteins form extracellular two-dimensional structures defining body and cell shape in *Drosophila melanogaster*. Open Biol..

[106] Hales K.G., Fuller M.T. (1997). Developmentally Regulated Mitochondrial Fusion Mediated by a Conserved, Novel, Predicted GTPase. Cell.

[107] Meng H., Yamashita C., Shiba-Fukushima K., Inoshita T., Funayama M., Sato S., Hatta T., Natsume T., Umitsu M., Takagi J. (2017). Loss of Parkinson’s disease-associated protein CHCHD2 affects mitochondrial crista structure and destabilizes cytochrome c. Nat. Commun..

[108] De Gregorio E., Spellman P.T., Tzou P., Rubin G.M., Lemaitre B. (2002). The Toll and Imd pathways are the major regulators of the immune response in Drosophila. EMBO J..

[109] Zhong W., McClure C.D., Evans C.R., Mlynski D.T., Immonen E., Ritchie M.G., Priest N.K. (2013). Immune anticipation of mating in *Drosophila*: *Turandot M* promotes immunity against sexually transmitted fungal infections. Proc. R. Soc. B Boil. Sci..

[110] Subramanian M., Metya S.K., Sadaf S., Kumar S., Schwudke D., Hasan G. (2013). Altered lipid homeostasis in *Drosophila* InsP3 receptor mutants leads to obesity and hyperphagia. Dis. Model. Mech..

[111] Watkins P.A., Maiguel D., Jia Z., Pevsner J. (2007). Evidence for 26 distinct acyl-coenzyme A synthetase genes in the human genome. J. Lipid Res..

[112] Lee K.-A., Cho K.-C., Kim B., Jang I.-H., Nam K., Kwon Y.E., Kim M., Hyeon D.Y., Hwang D., Seol J.-H. (2018). Inflammation-Modulated Metabolic Reprogramming Is Required for DUOX-Dependent Gut Immunity in Drosophila. Cell Host Microbe.

[113] Wijffels G., Eisemann C., Riding G., Pearson R., Jones A., Willadsen P., Tellam R. (2001). A Novel Family of Chitin-binding Proteins from Insect Type 2 Peritrophic Matrix. J. Biol. Chem..

[114] Lu Y., Li Z. (2015). Notch signaling downstream target E(spl)mbeta is dispensable for adult midgut homeostasis in Drosophila. Gene.

[115] Voßfeldt H., Butzlaff M., Prüßing K., Chárthaigh R.-A.N., Karsten P., Lankes A., Hamm S., Simons M., Adryan B., Schulz J.B. (2012). Large-Scale Screen for Modifiers of Ataxin-3-Derived Polyglutamine-Induced Toxicity in Drosophila. PLoS ONE.

[116] Neely G.G., Hess A., Costigan M., Keene A.C., Goulas S., Langeslag M., Griffin R.S., Belfer I., Dai F., Smith S.B. (2010). A Genome-wide Drosophila Screen for Heat Nociception Identifies α2δ3 as an Evolutionarily Conserved Pain Gene. Cell.

[117] Hedengren M., Borge K., Hultmark D. (2000). Expression and Evolution of the Drosophila Attacin/Diptericin Gene Family. Biochem. Biophys. Res. Commun..

[118] Vig M., Peinelt C., Beck A., Koomoa D.L., Rabah D., Koblan-Huberson M., Kraft S., Turner H., Fleig A., Penner R. (2006). CRACM1 is a plasma membrane protein essential for store-operated Ca^2+^ entry. Science.

[119] Kongton K., McCall K., Phongdara A. (2014). Identification of gamma-interferon-inducible lysosomal thiol reductase (GILT) homologues in the fruit fly Drosophila melanogaster. Dev. Comp. Immunol..

[120] Verleyen P., Baggerman G., D’hertog W., Vierstraete E., Husson S.J., Schoofs L. (2006). Identification of new immune induced molecules in the haemolymph of Drosophila melanogaster by 2D-nanoLC MS/MS. J. Insect Physiol..

[121] Ertürk-Hasdemir D., Broemer M., Leulier F., Lane W.S., Paquette N., Hwang D., Kim C.-H., Stöven S., Meier P., Silverman N. (2009). Two roles for the *Drosophila* IKK complex in the activation of Relish and the induction of antimicrobial peptide genes. Proc. Natl. Acad. Sci. USA.

[122] Linnemannstöns K., Ripp C., Honemann-Capito M., Brechtel-Curth K., Hedderich M., Wodarz A. (2014). The PTK7-Related Transmembrane Proteins Off-track and Off-track 2 Are Co-receptors for Drosophila Wnt2 Required for Male Fertility. PLoS Genet..

[123] Zhou F., Qiang K.M., Beckingham K.M. (2016). Failure to Burrow and Tunnel Reveals Roles for jim lovell in the Growth and Endoreplication of the Drosophila Larval Tracheae. PLoS ONE.

[124] Lerch S., Zuber R., Gehring N., Wang Y., Eckel B., Klass K.-D., Lehmann F.-O., Moussian B. (2020). Resilin matrix distribution, variability and function in Drosophila. BMC Biol..

[125] Syed Z.A., Härd T., Uv A., van Dijk-Härd I.F. (2008). A Potential Role for Drosophila Mucins in Development and Physiology. PLoS ONE.

[126] Lung O., Wolfner M. (2001). Identification and characterization of the major Drosophila melanogaster mating plug protein. Insect Biochem. Mol. Biol..

[127] Campos I., A Geiger J., Santos A.C., Carlos V., Jacinto A. (2010). Genetic Screen in *Drosophila melanogaster* Uncovers a Novel Set of Genes Required for Embryonic Epithelial Repair. Genetics.

[128] Schnakenberg S.L., Matias W.R., Siegal M.L. (2011). Sperm-Storage Defects and Live Birth in Drosophila Females Lacking Spermathecal Secretory Cells. PLoS Biol..

[129] Lake C.M., Nielsen R.J., Bonner A.M., Eche S., White-Brown S., McKim K.S., Hawley R.S. (2019). Narya, a RING finger domain-containing protein, is required for meiotic DNA double-strand break formation and crossover maturation in Drosophila melanogaster. PLoS Genet..

[130] Paine-Saunders S., Fristrom D., Fristrom J.W. (1990). The Drosophila IMP-E2 gene encodes an apically secreted protein expressed during imaginal disc morphogenesis. Dev. Biol..

[131] Akhmetova K., Balasov M., Chesnokov I. (2021). Drosophila STING protein has a role in lipid metabolism. eLife.

[132] Goto A., Okado K., Martins N., Cai H., Barbier V., Lamiable O., Troxler L., Santiago E., Kuhn L., Paik D. (2018). The Kinase IKKβ Regulates a STING- and NF-κB-Dependent Antiviral Response Pathway in Drosophila. Immunity.

[133] Sadanandan S.A., Ekström J.-O., Hultmark D. (2016). Drosophila Melanogaster Transcriptional Response to Nora Virus Infection. https://umu.diva-portal.org/smash/get/diva2:1045375/FULLTEXT01.pdf.

[134] Carpenter J., Hutter S., Baines J.F., Roller J., Saminadin-Peter S.S., Parsch J., Jiggins F.M. (2009). The Transcriptional Response of Drosophila melanogaster to Infection with the Sigma Virus (Rhabdoviridae). PLoS ONE.

[135] Liao J.-F., Wu C.-P., Tang C.-K., Tsai C.-W., Rouhová L. (2019). Identification of Regulatory Host Genes Involved in Sigma Virus Replication Using RNAi Knockdown in Drosophila. Insects.

[136] Chtarbanova S., Lamiable O., Lee K.-Z., Galiana D., Troxler L., Meignin C., Hetru C., Hoffmann J.A., Daeffler L., Imler J.-L. (2014). Drosophila C Virus Systemic Infection Leads to Intestinal Obstruction. J. Virol..

[137] Cohen L., Moran Y., Sharon A., Segal D., Gordon D., Gurevitz M. (2009). Drosomycin, an Innate Immunity Peptide of Drosophila melanogaster, Interacts with the Fly Voltage-gated Sodium Channel. J. Biol. Chem..

[138] Chakrabarti S., Dudzic J.P., Li X., Collas E.J., Boquete J.-P., Lemaitre B. (2016). Remote Control of Intestinal Stem Cell Activity by Haemocytes in Drosophila. PLoS Genet..

[139] A Callus B., Mathey-Prevot B. (2002). SOCS36E, a novel Drosophila SOCS protein, suppresses JAK/STAT and EGF-R signalling in the imaginal wing disc. Oncogene.

