# Supplementary material for: Viruses in Laboratory Drosophila and Their Impact on Host Gene Expression
Source: Viruses. 2023 Aug 31;15(9):1849. doi: 10.3390/v15091849 (PMC10537266; doi:10.3390/v15091849)
Supplement: Supplementary file 1 [file viruses-15-01849-s001.zip › Supplementary_Files/Figure S1.pdf]

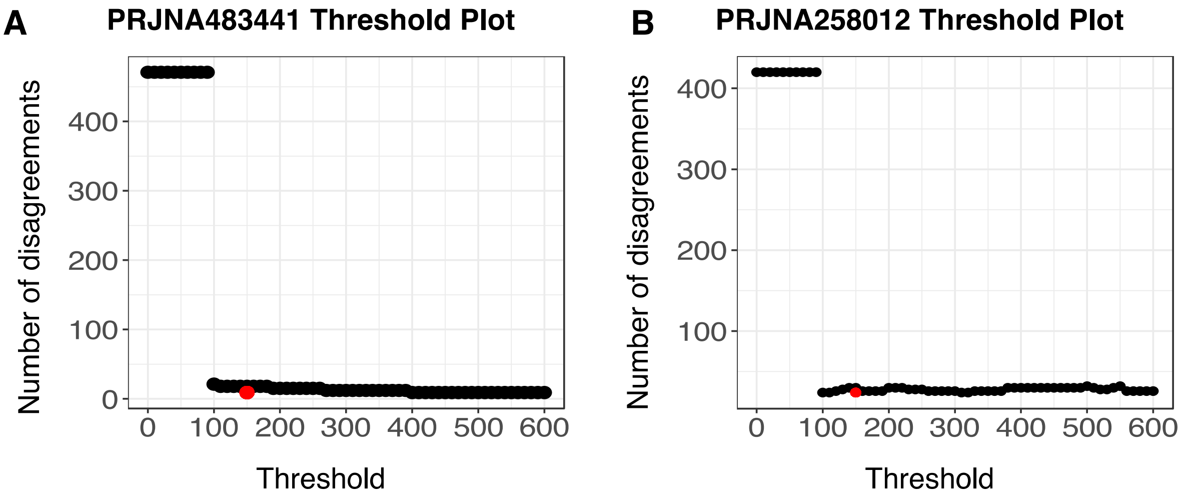

Figure S1 Best threshold to estimate viral presence

The datasets PRJNA483441 (**A**) and PRJNA258012 (**B**) have duplicate libraries. We compared the number of duplicates where there were disagreements in virus infection status when different threshold values from 0 to 600 were used i.e., the number of duplicates where one has present and the other has absent for a particular virus. The threshold at 150 is coloured red.
