# Supplementary material for: Viruses in Laboratory Drosophila and Their Impact on Host Gene Expression
Source: Viruses. 2023 Aug 31;15(9):1849. doi: 10.3390/v15091849 (PMC10537266; doi:10.3390/v15091849)
Supplement: Supplementary file 1 [file viruses-15-01849-s001.zip › Supplementary_Files/Figure S2 .pdf]

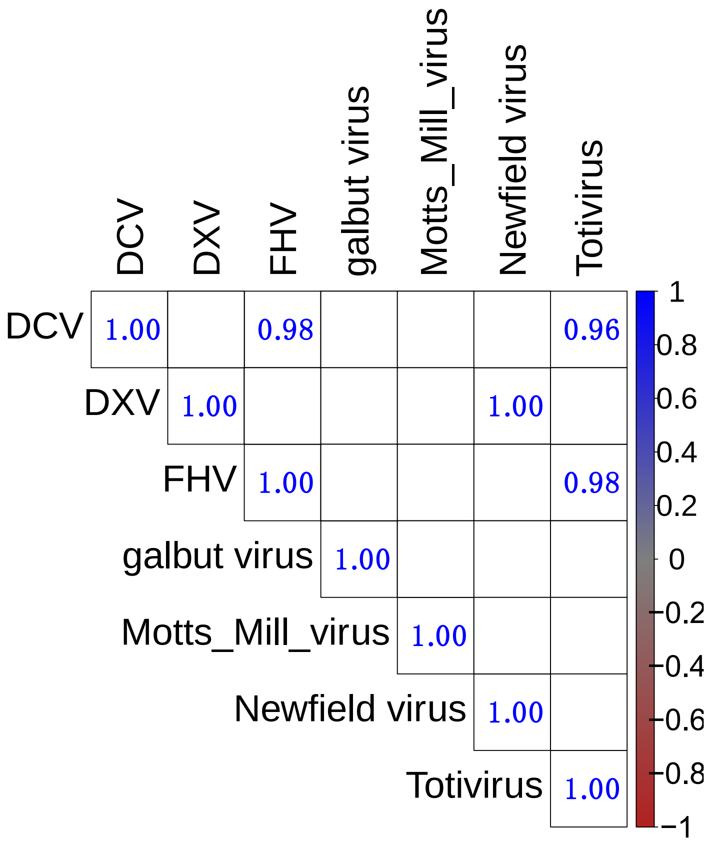

Figure S2 Correlations between normalised reads in PRJNA258012

The figure shows significant ($p < 0.001$) r values obtained via Spearman correlation between the normalised virus reads (RPKM) in samples in the dataset PRJNA258012. Blank squares have non-significant correlations.
