# Supplementary material for: Viruses in Laboratory Drosophila and Their Impact on Host Gene Expression
Source: Viruses. 2023 Aug 31;15(9):1849. doi: 10.3390/v15091849 (PMC10537266; doi:10.3390/v15091849)
Supplement: Supplementary file 1 [file viruses-15-01849-s001.zip › Supplementary_Files/Figure S4.pdf]

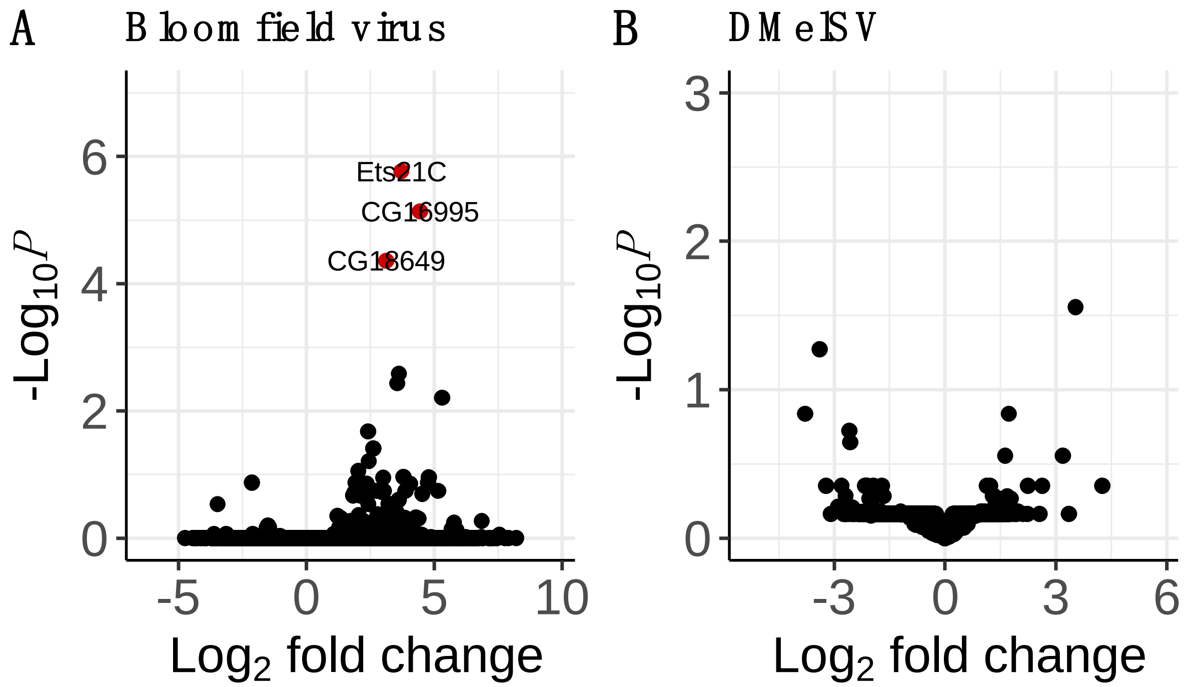

Figure S4 Effects of viral infection on host gene expression

Using uninfected females as baseline, the volcano plots show the effects of viral infection. The virus names and total number of variables (genes) used in each expression analysis is shown. None of the genes had significant changes in expression.
