# Supplementary figures and images for: Viruses in Laboratory Drosophila and Their Impact on Host Gene Expression
Source: Viruses. 2023 Aug 31;15(9):1849. doi: 10.3390/v15091849 (PMC10537266; doi:10.3390/v15091849)

**A) DAV**

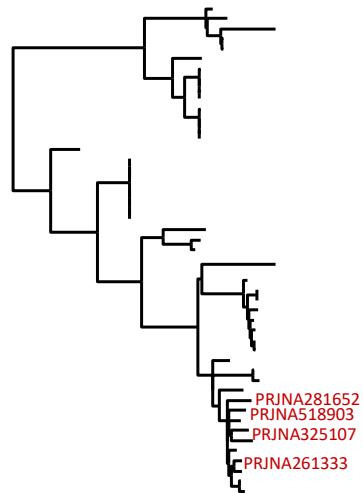

**B) Nora Virus**

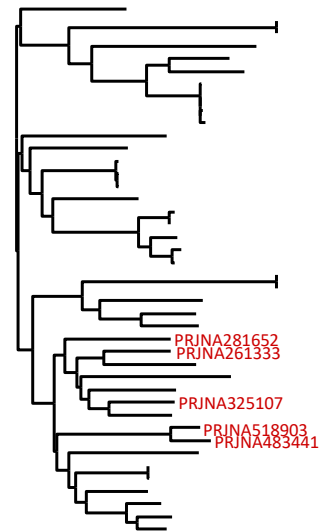

**C) DCV**

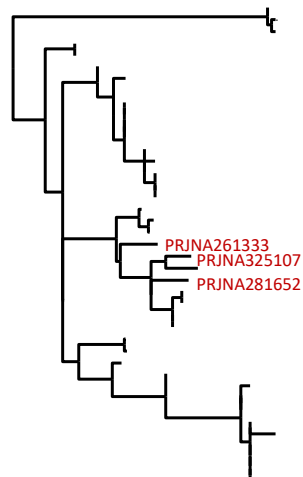

**D) Thika virus**

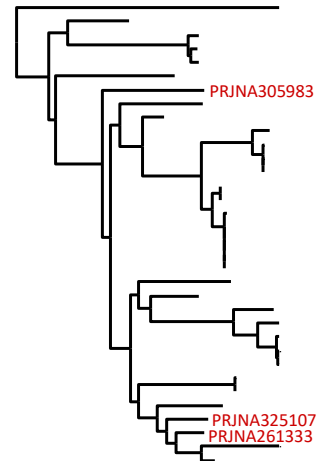

Supplement: Supplementary file 1 [file viruses-15-01849-s001.zip › Supplementary_Files/Figure S3.pdf]
